# Ka-Band Reflectarray with Cylindrical Dielectric Unit Cells: Optimized Additive Manufacturing and High-Permittivity Material Characterization

**DOI:** 10.3390/s25175480

**Published:** 2025-09-03

**Authors:** Michele Beccaria, Andrea Massaccesi, Mauro Lumia, Giuseppe Addamo, Angelo Freni, Paola Pirinoli

**Affiliations:** 1 Department of Electronics and Telecommunications, Politecnico di Torino, 10129 Turin, Italy; andrea.massaccesi@polito.it; 2 IEIIT, National Research Council, 10129 Torino, Italy; mauro.lumia@cnr.it (M.L.); giuseppe.addamo@cnr.it (G.A.); 3 Department of Information Engineering, University of Florence, 50139 Florence, Italy; angelo.freni@unifi.it

**Keywords:** antennas, high permittivity, reflectarray antennas, 3D-printing, additive manufacturing, mmWave communications, Zetamix ε

## Abstract

This paper discusses the design, manufacturing, and experimental characterization of a Ka-band fully dielectric reflectarray realized using Zetamix ε 7.5 ceramic material and additive manufacturing. Properly tuning the infill during the manufacturing process, it is possible to control the permittivity of the material, which can therefore be considered, to all intents and purposes, an additional degree of freedom for optimizing the unit cell and consequently the reflectarray performance. The optimal values of $\varepsilon_{r}$ are determined through numerical analysis of the unit cell and experimental characterization of bricks manufactured with different printing parameters. Then, the unit cell is used to design a medium-sized reflectarray with an aperture of 207.4$\;\lambda_{0}^{2}$ and a thickness of 0.44$\;\lambda_{0}$, at the design frequency $f_{0}=30\;$ GHz. The full-wave simulations of the designed RA and experimental measurements of a prototype confirm the excellent performance of the antenna, which exhibits a broadband flat response from 28 to 31 GHz and an aperture efficiency exceeding 50%.

## 1. Introduction

High-gain antennas are essential for modern microwave and millimeter-wave communication systems, including applications such as radar, satellite communications, remote sensing, point-to-point terrestrial links, and deep-space missions. Among others, Reflectarrays (RAs) [1,2] represent a well-assessed solution for realizing such antennas, possessing over other alternatives the advantages of being non-bulky, easy to manufacture, and low-cost.

Although the first RAs were designed assuming their realization with microstrip technology [3,4], alternative fabrication methods have been explored more recently [5,6,7,8,9,10,11,12,13,14,15,16]. A particularly effective solution consists of using dielectric resonator antennas (DRAs) as RA elements. DRAs [17,18,19,20] offer several advantages compared to printed elements, including wider bandwidths, higher radiation efficiency, and lower mutual coupling between elements. Their compatibility with Additive Manufacturing (AM) technologies, also known as 3D printing, provides greater design flexibility and cost-efficiency, enabling rapid prototyping and customized geometries.

The choice of technique adopted for antenna manufacturing has several effects on its features, mainly related to two different aspects. The first one is process accuracy, which is higher for Polymer Jetting (PJ) printers, allowing for the manufacturing of objects with many details, such as RAs working in the Ka band. In contrast, the materials that could be used with these printers are generally characterized by a low relative dielectric constant $\varepsilon_{r}$ and high losses. In contrast, printers based on the Fused Deposition Method (FDM) offer lower accuracy, but can work with a broader variety of dielectric materials with different properties. Recently, filaments with high relative permittivities have been introduced. These filaments are particularly suitable for the manufacturing of RF circuits and antennas, as they help to reduce their size [21,22,23,24,25,26,27,28]. Among them, there is Zetamix ε [29], a ceramic filament developed by *Nanoe* and available on the market in four different varieties, each characterized by a different value of relative permittivity varying between 2.2 and 10.

This work presents the design, fabrication, and experimental validation of a Ka-band dielectric RA, based on fully dielectric cylindrical Unit Cells (UCs) manufactured using Zetamix ε with $\varepsilon_{r}=7.5$. The novelty of this work lies not only in the design and experimental validation of the RA, but also in the rigorous characterization of the dielectric properties of Zetamix ε 7.5, which is fundamental when the porosity of the fused material compromises the high permittivity. The advantage of using a material with such a higher value for $\varepsilon_{r}$ is that, through the variation of the infill during the manufacturing process, it is possible to tune the final permittivity, adapting it to the application or changing it from one zone to another of the structure under construction [30]. Here, this nice feature has been exploited to optimize the UC and its performance by actually introducing a further degree of freedom in the unit cell design. According to the authors’ knowledge, this is the first time that the permittivity of the dielectric material has been used to optimize the performance of an RA unit cell, in addition to its geometrical parameters. The optimization process was carried out in two steps. First, the numerical analysis of the unit cell was performed for different values of the selected geometrical parameters and $\varepsilon_{r}$. Then, different bricks of the same material, obtained with different printing parameters and, in particular, different infills, were manufactured and experimentally characterized. Through this process, a permittivity close to the value determined through the numerical analysis was obtained and then used to design the reflectarray. Both its full-wave analysis and the experimental characterization of a medium-sized prototype show the good performance of the antenna and confirm the effectiveness of the proposed approach.

The paper is organized as follows: Section 2 summarizes the results of the numerical analysis of the unit cell, and experimental characterization of different blocks of Zetamix. The RA design procedure is described in Section 3 and the results of its characterization are discussed.

## 2. Unit Cell Characterization

To design a high-performance reflectarray, the unit cell must satisfy several properties, such as the ability to provide a reflection coefficient $S_{11}$ with magnitude as close to 0 dB as possible and phase that varies smoothly and possibly linearly over a range close to 360 degrees, a low dependence on the incident field’s angle of arrival and frequency. When, as in the present case, a dielectric cell is designed, its performance strongly depends on the material and the manufacturing techniques. The advantage of using a material with a high permittivity is that the optimal values for relative permittivity $\varepsilon_{r}$ can be obtained by appropriately controlling the infill [30]. For this reason, Zetamix ε 7.5 has been selected to enable the dielectric permittivity of the final structure to be tuned, keeping its value sufficiently high to reduce the UC dimensions as much as possible while maintaining the desired electromagnetic performance, contributing to the overall miniaturization and efficiency of the RA.

**Figure 1:**
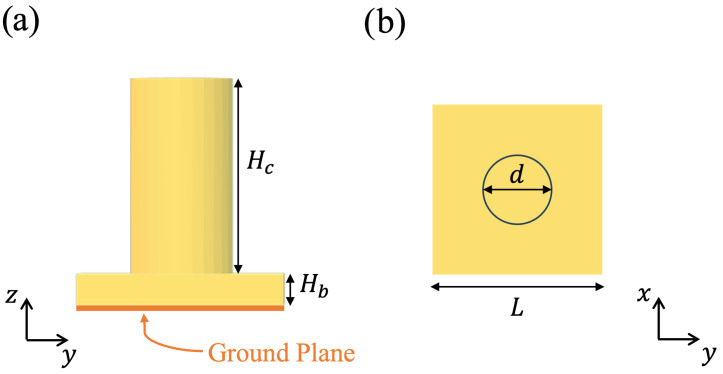
Geometry of the unit cell: (**a**) side view; (**b**) top view.

The geometry considered here for the UC is sketched in Figure 1. A square, uniform layer of dielectric material is deposited with thickness $H_{b}=0.6$ mm above a metallic ground plane. On top of the uniform layer, a cylindrical pillar with diameter *d* and height $H_{c}$ is printed in the center of the cell. For the electromagnetic characterization of the UC, this is considered embedded in a periodic lattice with periodicity equal to its dimension L=4 mm. Note that the periodicity is slightly less than $0.42\;\lambda_{0}$ at the upper end of the considered frequency band, which ranges from 28 GHz to 31 GHz. A full-wave periodic analysis was performed in the frequency domain by using CST Microwave Studio™ (CST 2024). Orthogonal and skew plane wave incidences were considered.

The diameter *d* of the pillar is varied to control the phase of the reflection coefficient $S_{11}$, while its height $H_{c}$ and the relative permittivity $\varepsilon_{r}$ are used to optimize the UC performance and reduce its size. Three different analyses were performed: first, the height of the pillar was kept fixed and the variation of the magnitude $|S_{11}|$ and phase $∠S_{11}$ of the reflection coefficient versus the diameter *d* was obtained for several values of $\varepsilon_{r}$. Then, the effects on the UC performance of the pillar height and the direction of arrival of the incident field were considered.

**Figure 2:**
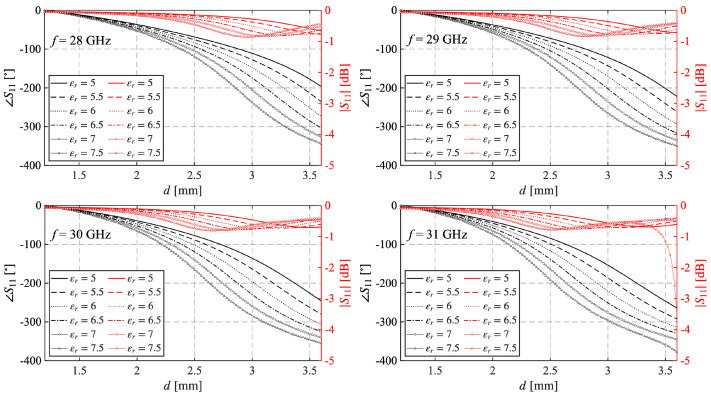
Variation of $|S_{11}|$ and $∠S_{11}$ for different values of $\varepsilon_{r}$ evaluated in correspondence of 4 frequencies, and inside the considered band $H_{c}=3.8$ mm.

Figure 2 plots the variation of the magnitude ($|S_{11}|$) and phase ($∠S_{11}$) of the reflection coefficient for different values of relative permittivity. Examining the curves associated with $∠S_{11}$ show that different values of $\varepsilon_{r}$ affect both the range of variation and frequency dispersion. In particular, increasing the value of $\varepsilon_{r}$ enlarges the phase range, whose minimum occurs for $\varepsilon_{r}=5$ at 28 GHz, and also helps to keep its frequency dependence lower. This behavior seems to favor higher values of permittivity. However, examining the curves related to the magnitude $|S_{11}|$ (red curves), it becomes apparent that a second resonance, accompanied by a strong attenuation, appears for $\varepsilon_{r}=7.5$ at 31 GHz, and therefore, it is advisable to discard that value for $\varepsilon_{r}$.

**Figure 3:**
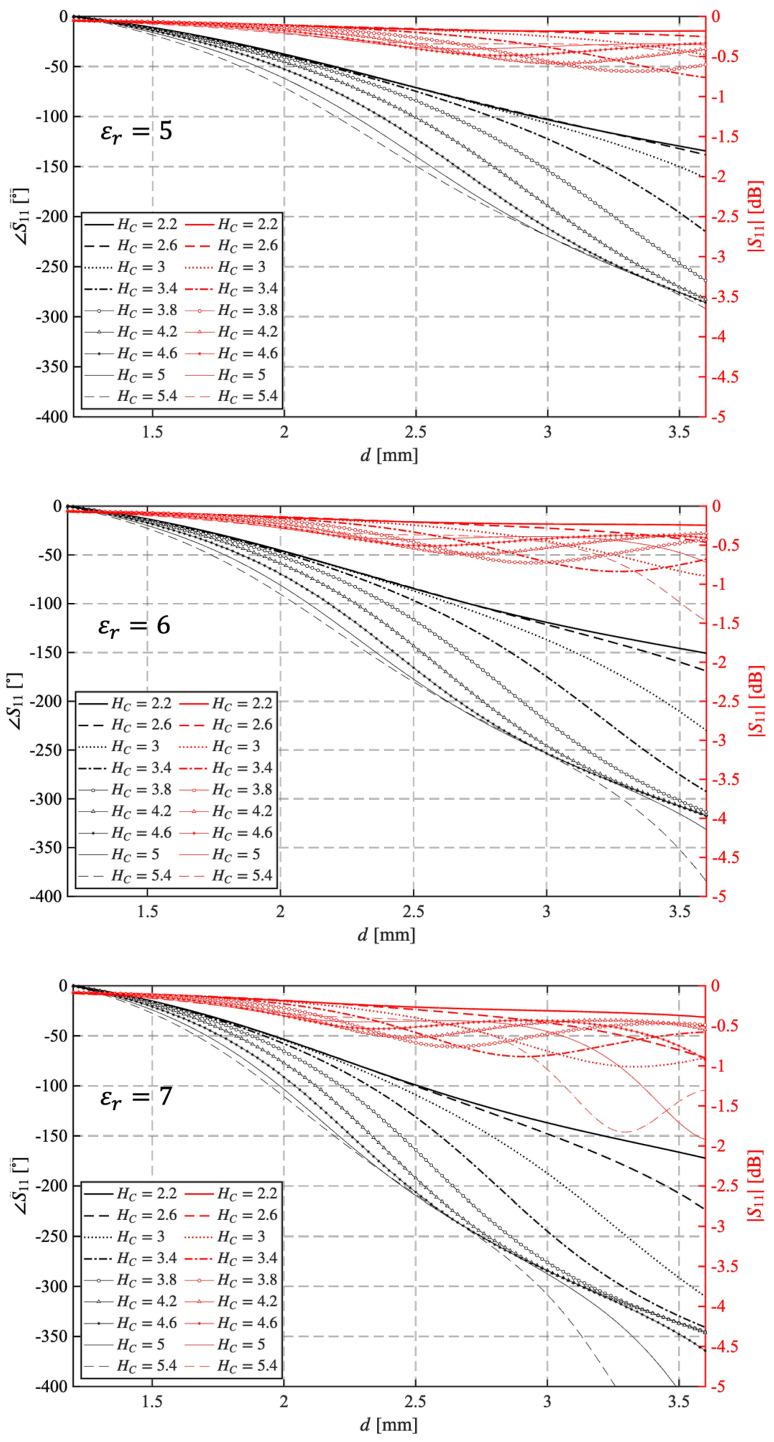
Variation of $|S_{11}|$ and $∠S_{11}$ for different values of $H_{c}$ evaluated for $\varepsilon_{r}=5,6,7$ at 31 GHz.

The second analysis is focused on the evaluation of the effect of pillar height $H_{c}$ on UC performance. Figure 3 shows the variation of $|S_{11}|$ and $∠S_{11}$ versus *d*, evaluated at 31 GHz, to check if other resonances occur. Each plot shows results for a different value of $\varepsilon_{r}$, while the curves refer to different values of $H_{c}$. As expected, increasing the height of the pillar increases the phase delay dynamics provided by the unit cell. When $H_{c}=5.4$ mm, the phase range varies from approximately 280° for $\varepsilon_{r}=5$ to a range greater than 400° for $\varepsilon_{r}=7$. However, since the unit cell usually does not cover the full 360° range, and a reasonable value to ensure an acceptable phase error on the RA surface is approximately 300°, the plots in Figure 3 show that this range is never reached with the considered set of $H_{c}$ values when $\varepsilon_{r}=5$. Conversely, this range is provided by a pillar with $H_{c}=3.8$ mm and $\varepsilon_{r}=6$ or $H_{c}=3$ mm and $\varepsilon_{r}=7$. Concerning $|S_{11}|$, it never drops below −2 dB in any of the considered cases, even when the relative permittivity is $\varepsilon_{r}=7$ and a second resonance appears at the highest $H_{c}$ values.

**Figure 4:**
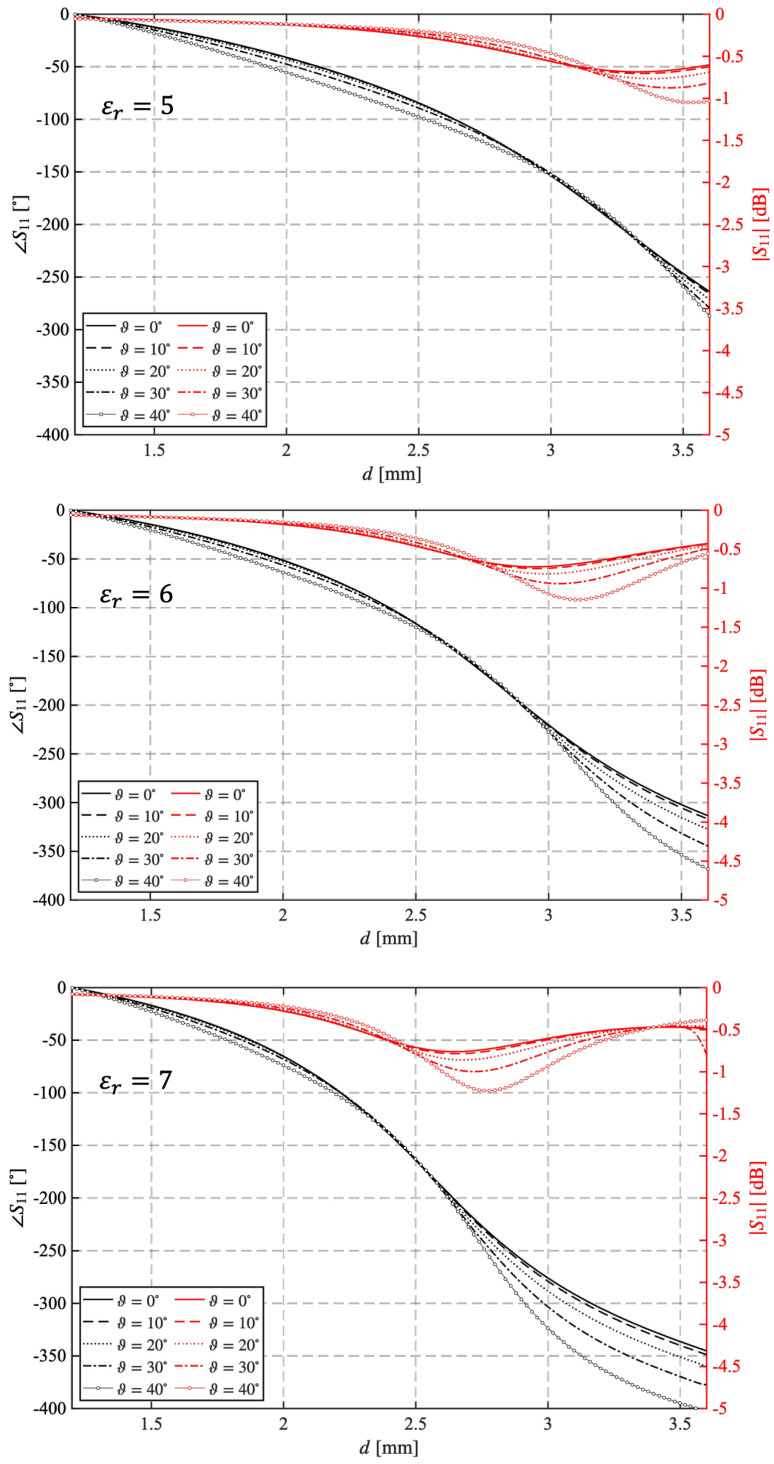
Variation of $|S_{11}|$ and $∠S_{11}$ for different values of the angle of incidence evaluated for $H_{c}=3.8\;$ mm and $\varepsilon_{r}$ = 5, 6, 7 at 31 GHz.

Finally, the curves in the three plots of Figure 4 show the effect of the angle of incidence of the incoming field on the behavior of the unit cell made of a dielectric material with relative permittivity equal to 5, 6, or 7. The results have been obtained at 31 GHz, with $H_{c}=3.8\;$ mm. In contrast to the previous results, the optimal value for $\varepsilon_{r}$ in this case is 5, since the dependence on the direction of arrival of the incident field is minimal.

Based on these results, it was decided to select a relative permittivity of 6 and $H_{c}=3.8\;$ mm, as they appear to represent the best compromise between the unit cell’s performance and its size.

### 3D Printing Parameters Assessment and Material Experimental Characterization

Having fixed the optimal value for $\varepsilon_{r}$, it was necessary to assess the printer parameters to obtain it. To achieve this aim, various brick samples, with nominal dimensions 8.64 mm × 4.32 mm × 3.5 mm, have been manufactured using different configurations of the printing parameters as explained later. The fabrication of the bricks was performed using a Raise3D Pro3 printer [31], the same adopted for the manufacturing of the reflectarray. The nominal dimensions of the bricks match exactly the internal dimensions of the WR34 waveguide used for the permittivity evaluation.

**Figure 5:**
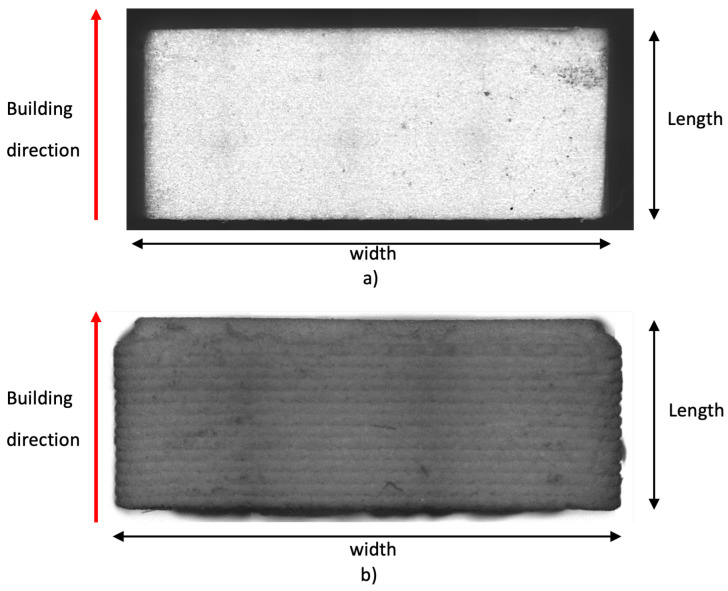
Detailed view of the WR34 samples along the building direction. (**a**) Sample 1 of the first set, manufactured using a typical printer setup. (**b**) Sample 1 of the second set, manufactured with values of the printer parameters adjusted for the specific case.

Initially, standard printing parameters within the range provided by *Nanoe* for Zetamix were used. They are summarized in column 2 of Table 1. A first set of three bricks was manufactured using these parameters and experimentally characterized. A CNC optical measuring system has been employed to investigate their mechanical structure and determine their dimensions. Figure 5a shows a detailed view of a brick sample along the building direction, confirming that the brick exhibits a relatively uniform material distribution due to the 100% infill. However, there are slight variations in size among the bricks, as indicated by the values in Table 2. It is important to note that the samples do not completely fill the WR34 waveguide. Therefore, to eliminate any uncertainties regarding their position, the samples were consistently placed at one corner of the waveguide.

**Figure 6:**
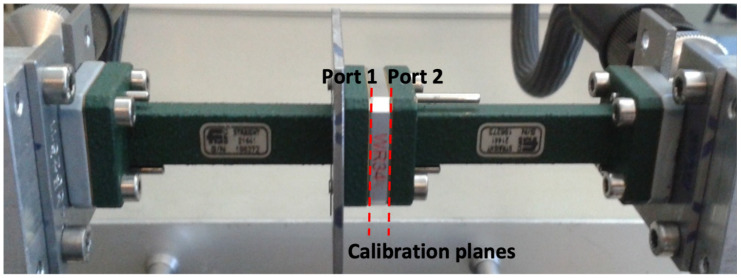
Measurement setup.

The electromagnetic characterization of the samples was carried out by filling the sample into a WR34 waveguide, which was connected to the Vector Network Analyzer (VNA) through a pair of coaxial waveguide launchers. A classical through-reflection-line (TRL) technique has been exploited for accurate calibration. Figure 6 shows the measurement setup and the calibration planes. The material’s characterization in terms of complex dielectric constant is derived by best fitting between the simulated and measured reflection and transmission coefficients.

**Figure 7:**
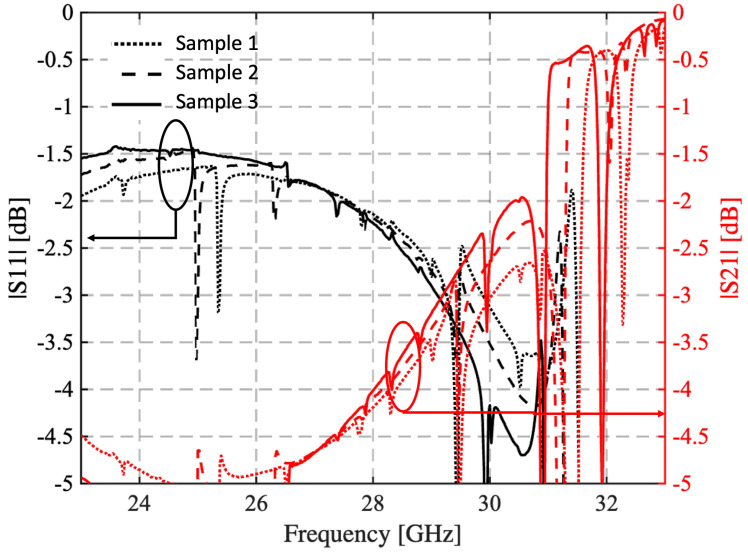
Measured reflection and transmission coefficient of the three samples manufactured with the setup suggested by *Nanoe* and considering 100% infill. The curves in black refer to the reflection coefficient, while those in red show the transmission coefficient.

It is well known that this procedure is quite effective when a single-mode operation can be exploited and the transmission curves present a significant peak. This can be generally emphasized by exploiting two irises [32]. Unfortunately, the high relative permittivity of the Zetamix samples on one side and the non-perfect dimensions of the samples on the other make the cited procedure much more complex. The measured curves, reported in Figure 7, present indeed different minima/peaks. They are related to the excitation of the higher-order modes ($TE_{m,0}$, with *m* = 2, 3…) in the loaded region. Moreover, the set of sample curves exhibits excellent dispersion due to dimensional variability. Despite these considerations, it would be possible to fix the relative permittivity to a value of around 7.6 with a dielectric loss tangent $\tan\delta =5\times 10^{-3}$.

To target a relative permittivity close to $\varepsilon_{r}=6$, the guidelines provided by *Nanoe* were followed. In particular, in [30] a plot showing the variation of the equivalent $\varepsilon_{r}$ versus the material porosity suggests setting the infill density to approximately 90%. This choice was combined with a tuning of the printing parameters (nozzle temperature, bed temperature, and speed), as reported in Table 1, to ensure both electromagnetic performance and successful manufacturability of the entire structure. The printing temperature was set to 285 °C with a heated bed temperature of 110 °C for proper adhesion. The extrusion width was 0.4 mm, with a layer height of 0.2 mm and a first layer height of 0.4 mm to enhance the foundation’s stability. To ensure uniform dielectric density and minimize inhomogeneities, an infill density of 90% with a gyroid pattern was used, providing consistent internal support and an optimized weight-to-strength ratio. A flow rate of 100% was maintained to minimize under-extrusion and ensure consistent density throughout the printed elements. The first layer was printed at 7 mm/s for better adhesion, while the subsequent layers were printed at 17 mm/s. To maintain a stable thermal environment, cooling was completely disabled (i.e., 0% fan speed). In addition, a brim of 12 lines was used to enhance bed adhesion and prevent warping.

One of the three samples obtained with this printer setup is shown in Figure 5b, and their sizes are summarized in Table 3. As can be seen in Figure 5b, this sample is less uniform than the one in Figure 5a. However, the size of the different bricks is more stable, as shown in Table 3, even if it is slightly lower than the waveguide dimensions.

**Figure 8:**
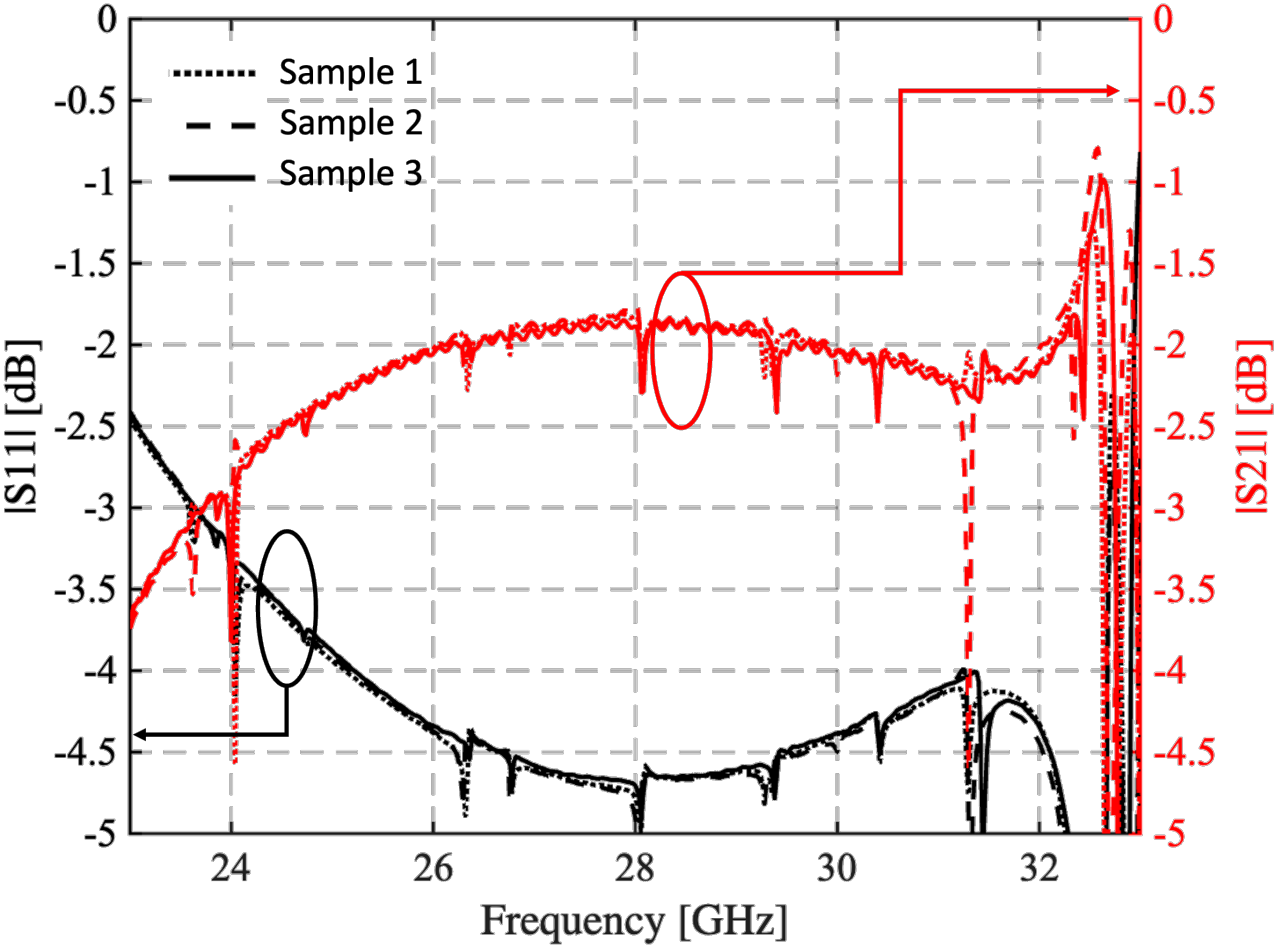
Measured reflection and transmission coefficient of the three samples manufactured with the defined setup. The black curves refer to the reflection coefficient, while those in red show the transmission coefficient.

The results of the brick measurements are reported in Figure 8, where the frequency behavior of the reflection coefficient and the transmission coefficient is plotted. In this case, the set of blocks exhibits good repeatability, and the spikes due to higher-order mode resonances are less pronounced. The samples’ relative permittivity is confirmed to be around 5.9, which is very close to the desired value, while $\tan\delta =2\times 10^{-3}$.

## 3. Reflectarray Antenna Design and Experimental Validation

### 3.1. UC Performance

In the previous section, the optimisation process and the results of the experimental characterisation of the material that led to the definition of the unit cell parameters were described. The final values for the UC geometrical parameters are: L=4 mm, $H_{b}=0.6\;$ mm, and $H_{c}=3.8\;$ mm, while the diameter of the cylindrical resonator, *d*, is varied in the range of 1.2mm≤d≤3.6mm to ensure the UC manufacturing still keeping a good range of variation for the phase of $S_{11}$. The design frequency is $f_{0}=30\;$ GHz, and the UC performance is tested over the band 28 GHz–31 GHz.

**Figure 9:**
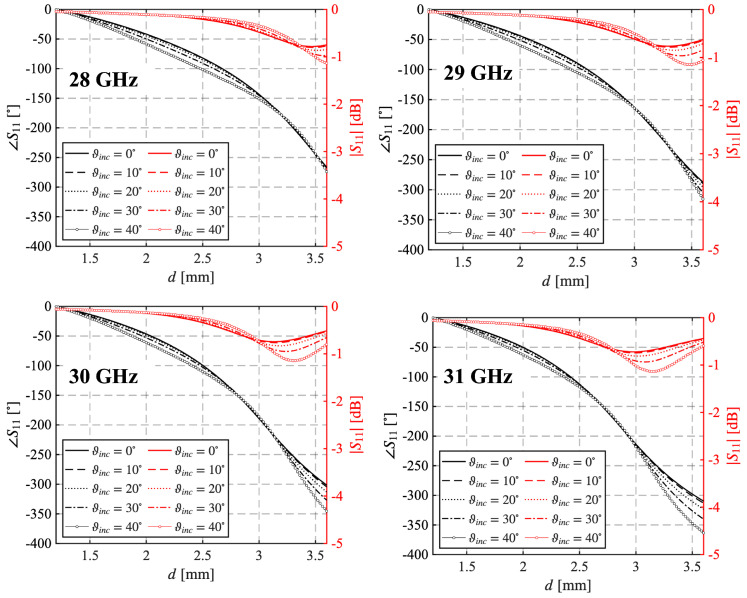
Phase and magnitude of the reflection coefficient $S_{11}$ as a function of the diameter *d* for different frequencies computed for many angles of arrival.

Since the experimental characterization of the material returns a value for $\varepsilon_{r}=5.9$ equal to none of those considered in the UC optimization process, its Floquet analysis was carried out one more time, and the plots in Figure 9 were obtained. They show the phase $∠S_{11}$ and magnitude $|S_{11}|$ of the reflection coefficient versus *d* for different angles of incidence $\vartheta_{\mathrm{inc}}=0{}^{\circ}-40{}^{\circ}$ and several frequencies in the band of interest. As expected, the phase response exhibits a smooth and monotonic variation with increasing diameter *d*, providing a wide phase tuning range. At the design frequency, the phase range covers 301°, which is sufficient for the scope of the work, as already highlighted. Moreover, the phase curves corresponding to different incidence angles are nearly parallel, indicating a weak dependence on the angle of incidence. As concerns the magnitude $|S_{11}|$, it remains above −1.1 dB throughout the *d* variation range, indicating high reflection efficiency. Furthermore, the curves exhibit a consistent trend across all the frequencies evaluated, revealing the wide-band behavior of the UC. This frequency-independent response highlights the robustness of the design, ensuring stable performance over a broad operational bandwidth.

### 3.2. RA Design and Experimental Characterization

Using the unit cell described above, a reflectarray operating at the center frequency $f_{0}=30\;\;\mathrm{GHz}$ has been designed.

**Figure 10:**
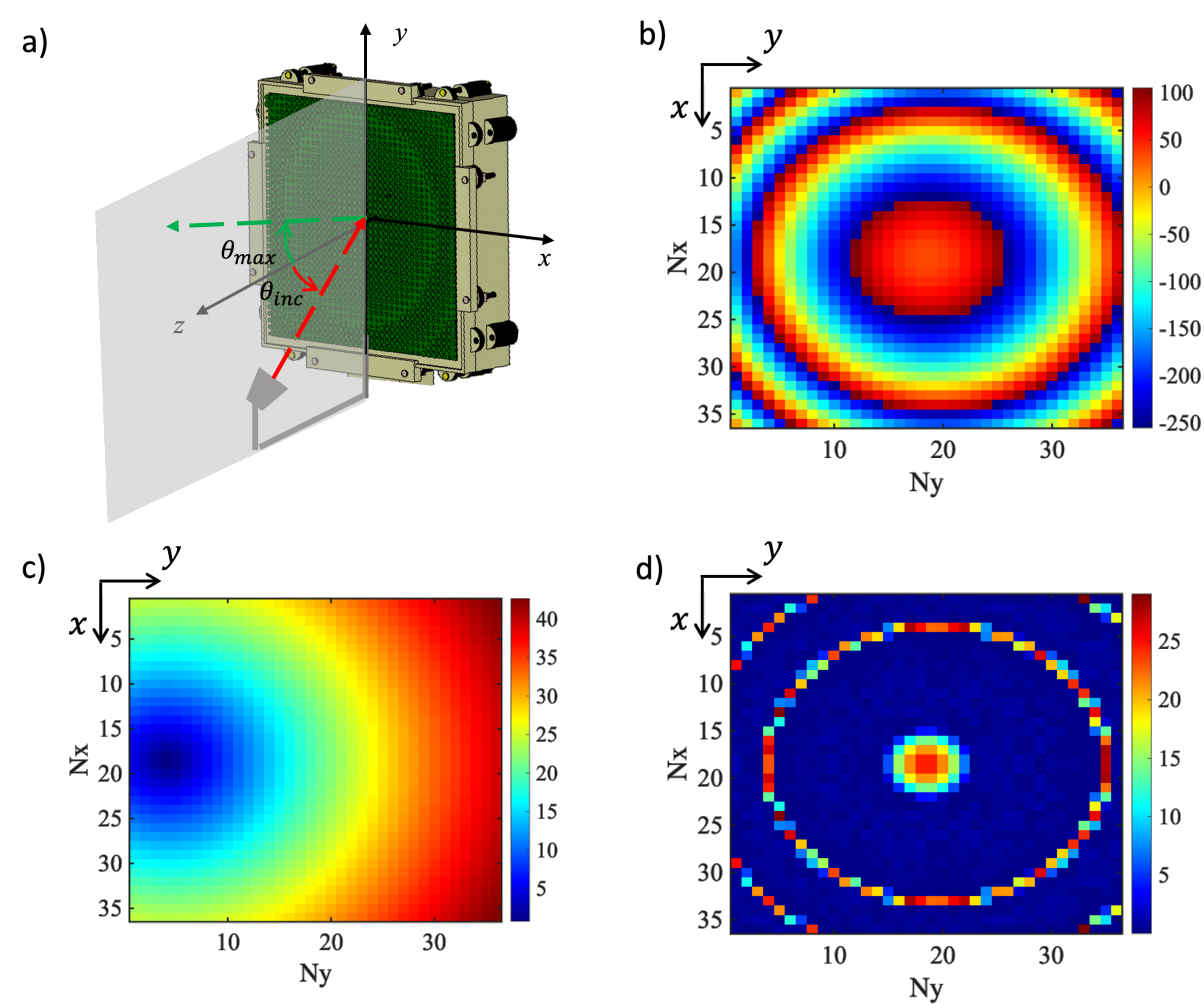
(**a**) Sketch of the RA geometry with the coordinate system superimposed. (**b**) Required phase delay distribution for the designed RA at $f_{0}=30\;$ GHz. (**c**) Incidence angle map across the RA surface with respect to its normal vector. (**d**) Phase error map showing the residual discrepancies between the ideal and implemented phase distribution at $f_{0}=30\;$ GHz.

In Figure 10a, the reflectarray geometry with the adopted coordinate system is sketched. The reflecting squared surface consists of 36×36 UCs and has a side $D=144\;\mathrm{mm}=14.4\;\lambda_{0}$ at $f_{0}$. The RA is configured in an offset arrangement and the feeder is the circular horn introduced in [33], whose radiation pattern can be modeled as a function of $\cos^{q}\left(\theta \right)$ with q=12.5, and is located at a focal distance of 168 mm from the center of the RA. Both the incidence and the direction of maximum radiation are in the vertical plane (the yz plane in the coordinate system in Figure 10a). The incidence is characterized by angles $\left(\theta_{\mathrm{inc}},\varphi_{\mathrm{inc}}\right)=\left(20{}^{\circ},270{}^{\circ}\right)$, while the reflectarray is designed to point the reflected field in a direction characterized by $\theta_{max}=20{}^{\circ}$ and $\varphi_{max}=90{}^{\circ}$.

The required phase delay distribution is plotted in Figure 10b, calculated to compensate for the phase of the incident field on the RA surface, represented in Figure 10c. The phase error distribution, illustrated in Figure 10d, highlights the residual deviations between the required phase response and the one provided by the unit cell, which does not cover the entire range of 360°, as already pointed out. From this, it emerges that the error introduced in the phase delay is, for most of the unit cell, lower than 5°, and also in the worst case it is never larger than 30°, and this confirms the proper design of the unit cell.

**Figure 11:**
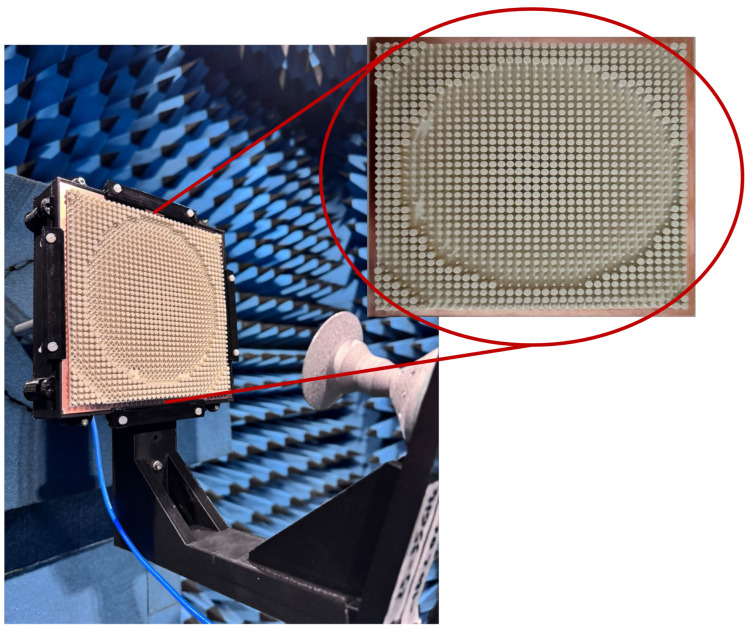
Setup of the manufactured RA prototype in the anechoic chamber. Inset: front view of the RA prototype.

First, the RA was numerically analyzed using the time-domain solver of CST Microwave Studio™. A prototype was then manufactured and experimentally characterized. Figure 11 shows the manufactured RA, mounted inside the anechoic chamber at Politecnico di Torino, where the measurements were conducted. The antenna was tested in the spherical near-field test range, using an angular sampling of 1° in both azimuth and elevation [34]. The near-field data were post-processed to obtain the far-field radiation patterns. The gain was estimated by comparing the measured field level of the reflectarray with that of a reference standard horn. The aperture efficiency was then computed as the ratio between the measured gain and the theoretical directivity of a uniformly illuminated aperture having the same physical size. The inset in Figure 11 displays a photo of the front view of the prototype.

**Figure 12:**
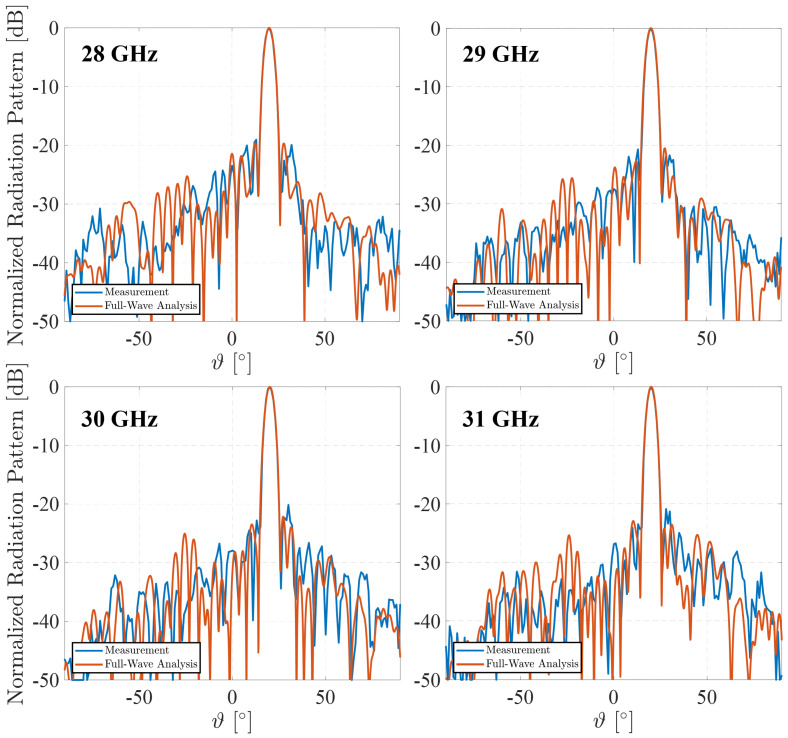
Simulated and measured co-polar radiation patterns in the E-plane.

**Figure 13:**
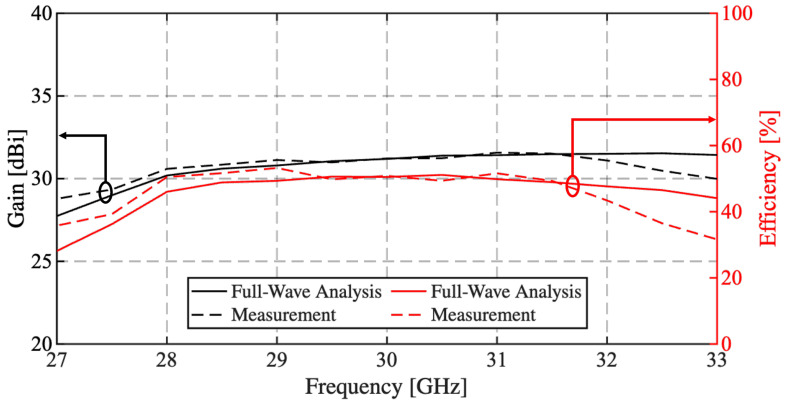
Measured and simulated gain and aperture efficiency variation as a function of frequency.

The principal results of the numerical and experimental characterization are summarized in Figure 12 and Figure 13. Figure 12 shows the normalized radiation patterns in the E-plane (vertical plane) of the manufactured RA at 28 GHz–31 GHz, compared with those obtained with the RA full-wave analysis. The main beam is successfully steered towards $\theta_{\max}=20{}^{\circ}$, as intended by the design specifications. The main lobe exhibits excellent agreement between measurements and simulations, validating the RA’s ability to achieve precise beam pointing. Minimal discrepancies can be observed, which can be primarily attributed to two factors: fabrication tolerances inherent in the 3D printing process and alignment inaccuracies during the measurement setup in the anechoic chamber. Despite these slight variations, the RA maintains a stable radiation pattern across the frequency range, demonstrating good frequency robustness with well-controlled side lobe levels (SLLs), consistently lower than −20 dB.

Figure 13 shows the measured and simulated gain and aperture efficiency of the RA as functions of the frequency. The measured gain has a peak value of 31.6 dBi at 31 GHz. It exhibits a remarkably flat response within the considered frequency range of 28 GHz to 31 GHz, with a measured 1-dB bandwidth of 16%, highlighting the robust broadband performance of the RA. The measured aperture efficiency exceeds 50% throughout the band, reaching a maximum of 53.2% at 29 GHz.

Overall, the results validate the effectiveness of the proposed design approach, demonstrating excellent agreement between full-wave simulations and experimental data. Moreover, they confirm that the use of a material such as Zetamix ε, with a tunable value of equivalent $\varepsilon_{r}$ introduces an additional degree of freedom that can be used to optimize the performance of the device to be built, as in this case the RA.

Finally, Table 4 summarizes the comparison with 3D-printed dielectric reflectarrays reported in the literature, spanning from 2017 to 2024 [24,25,26,27,28]. It can be observed that the solution in [28] exhibits the highest aperture efficiency. However, the RA is quite small and is center-fed, while the proposed one has an offset feed. The designs in [24,25,26,27] show dimensions larger than those in [28] with efficiencies below 35%. In contrast, the proposed RA combines a larger electrical aperture of $207.4\;\lambda_{0}^{2}$ with a compact thickness of $0.44\;\lambda_{0}$ and a measured gain above 30.5 dBi in the 16% 1-dB gain bandwidth, while maintaining an aperture efficiency above 50%. Regarding the concerns with the 1-dB gain bandwidth, the largest one is in [25] and comes at the cost of a lower gain. The solutions in [26,28] show a slightly larger bandwidth compared with the proposed RA, but it must be taken into account that their reflectarrays’ dimensions, and therefore the gain, are much smaller (see columns 4 and 6 of Table 4). This performance can be explained by considering that the unit cell presents an almost linear variation of the phase delay versus its geometrical dimensions, a significantly reduced sensitivity with respect to the incidence angle of the impinging plane wave, and a uniform global phase shift with the frequency (i.e., the phase delay curves versus *d* are almost parallel for different frequencies). These properties enable us to achieve a reasonable efficiency and bandwidth, albeit with the requirement for high gain and offset-center illumination.

## 4. Conclusions

This work focuses on the design, manufacturing, and experimental validation of a fully dielectric RA operating in the Ka band that exploits Zetamix ε 7.5 ceramic-based material through additive manufacturing. The RA uses cylindrical UCs, with optimized 3D printing parameters tailored to address the unique challenges posed by the high-permittivity material. The possibility to control the value of the equivalent relative permittivity by varying the infill during the manufacturing process allows us to consider $\varepsilon_{r}$ as a further degree of freedom that can be exploited to optimize the unit cell and consequently the RA performance. The excellent agreement between the full-wave analysis and the experimental results confirms the effectiveness of the proposed approach.

## Figures and Tables

**Table 1:** Comparison between recommended and adopted printing parameters for Zetamix ε 7.5.

Parameter	Recommended Values [30]	Used Values
Printing temperature	290 °C	285 °C
Plate temperature	110 °C	110 °C
Nozzle size	0.4 mm, 0.6 mm	0.4 mm
Layer thickness	0.2 mm	0.2 mm
First layer printing speed	2.5 mm/s	7 mm/s
General printing speed	15 mm/s	17 mm/s
Cooling	0% (remove fan)	0% (fan disabled)
Infill density	100% (Gyroid pattern)	90% (Gyroid pattern)
Flow rate	100%	100%
Retraction speed	-	40 mm/s

**Table 2:** Dimensions of the samples manufactured with the standard printer setup.

Sample	Width (mm)	Height (mm)	Length (mm)
1	8.64	4.24	3.50
2	8.62	4.26	3.52
3	8.58	4.30	3.52

**Table 3:** Dimensions of the samples manufactured with the defined setup.

Sample	Width (mm)	Height (mm)	Length (mm)
1	8.61	4.28	3.48
2	8.62	4.29	3.48
3	8.61	4.28	3.50

**Table 4:** Comparison with recent state-of-the-art Ka-band dielectric reflectarrays fabricated via additive manufacturing.

Ref.	Freq. [GHz]	$\varepsilon_{r}$	Area ($\lambda_{0}^{2}$)	Thickness ($\lambda_{0}$)	Gain [dBi]	Ap. Eff. [%]	1-dB BW [%]
[24]	30	4.4	140	0.65	28	31	12
[25]	35	10.2	153.86	0.148	≈24.8	17.7	23
[26]	30	2.8	129.3	≈3	≈25	22.8	17.5
[27]	27	7.5/2.2	≈119.6	≈1.52	27.22	35	3.33
[28]	28	4	25.4	0.56	23.8	75	16.7
**This** **Work**	30	5.9	207.4	0.44	31.2	50.8	16

## Data Availability

All data generated or analysed during this study are included in this article.
